# Notes and Notices

**Published:** 1893-08

**Authors:** 


### NOTES AND NOTICES.

THE next annual class for instruction in orificial surgery will assemble in Chicago on the morning of September 4th. It will have a four hours daily session during the week. For further particulars address Dr. E. H. Pratt, room 56, Central Music Hall, Chicago.

DR. J. W. THOMSON has removed to 155 West Forty-eighth Street, New York City.

DR. CHARLES P. BEAMAN, long located at Chattanooga, Tennessee, has removed to Ithaca, New York, where he has associated himself in partnership with Dr. Bishop, under the firm-name of Bishop & Beaman.

N. Y. STATE HOMŒOPATHIC HOSPITAL AT MIDDLETOWN.—By the recent action of the Commission in Lunacy, reducing the territory embraced in this hospital district to the four counties of Orange, Rockland, Sullivan, and Ulster, we are enabled to receive patients from any part of the State, both public and private, for whom homœopathic treatment is desired.

The friends of public patients residing out of this district are required to pay for their transportation from their homes to the hospital.

Private patients can be received from any part of the State.

NEW YORK HOMŒOPATHIC MEDICAL COLLEGE.—Dr. T. F. Allen, so long Dean of this College, has resigned, and Dr. William Tod Helmuth, M. D., LL. D., celebrated for his surgical skill, and as well for his literary ability, especially in poetry, has been elected to the vacant position. Our readers who have noticed the advertisement of this college for so many years upon the fourth page of the cover of this journal will observe also that Dr. George G. Shelton has been made Registrar.

HOMŒOPATHIC MEDICAL SOCIETY OF PENNSYLVANIA.

PHILADELPHIA, August 1st, 1893.

DEAR DOCTOR—Allow me to call your attention to the Twenty-ninth Session of the Homœopathic Medical Society of Pennsylvania, which will convene in Pittsburgh, on Tuesday, September 19th, 1893.

In fixing the date for this meeting, careful consideration has been given as to the best time for visiting the World's Fair at Chicago. The first part of cool September can be spent at the Fair, followed by a useful and enjoyable three days at Pittsburgh; or, if duty is preferred before pleasure, the Annual Meeting can be attended first.

I use the word "duty" intentionally and advisedly; for it certainly is the "duty" of each one of us to attend the Annual Sessions of our State Society. It is only by such meetings that we can learn both the Society's and our individual needs. Every one of us can singly do a great deal to help our Society and each other, and much more can be done by our united action. To this united action, above all else, was due our glorious success in gaining the Three Board Medical Examiners' Bill. The success attained in this Bill should act as a reward for our past endeavors; as an example of what our united and determined action can achieve; and, most important of all, as an encouraging stimulus for further aggression on our part. I also use the word "aggression" intentionally and advisedly. We have much, very, very much to do to win the place Homœopathy deserves, and ought to occupy in the medical profession both as a School of Medicine, and before the public throughout the United States.

Pennsylvania is a big State, is an important State—is the Keystone State! We have a large representation of physicians, hospitals, dispensaries, and the *first*, in every respect, Homœopathic Medical College in the world. Being thus big and strong, we can and ought to accomplish much. The good work we do in our State for Homœopathy and for the general public is reflected in other States and helps them to do what we have done.

Let every one then come to the Annual Meeting at Pittsburgh in September. Matters of general interest and benefit to every homœopathic physician will be there unfolded; and the remembrance of the warm hand-shake and genial "glad to see you" will lighten our labors during the coming year.

With kind regards,

Fraternally yours,

J. C. GUERNSEY, M. D.

